# An emotional roller coaster - family members’ experiences of being a caregiver throughout a cancer trajectory

**DOI:** 10.1080/17482631.2022.2137965

**Published:** 2022-10-20

**Authors:** Monica Solberg, Geir Vegard Berg, Hege Kristin Andreassen

**Affiliations:** a Department of Health Siences in Gjøvik, Norwegian University of Science and Technology, Trondheim, Norway; b Innlandet Hospital Trust, Norway; c Technoligy and the Artic University of Norway, Tromsø, Norway

**Keywords:** Family members, caregiver, longitudinal design, qualitative, in-depth interviews, experiences, cancer trajectory, cancer care

## Abstract

**Purpose:**

To explore family members’ experiences of caregiving throughout a cancer trajectory from diagnosis until around one year after chemotherapy and radiation treatment ended.

**Method:**

We conducted a longitudinal qualitative study using in-depth interviews with 13 family members at one to three points of time: before, during, and after treatment. To analyse the interviews, we leaned on Braun and Clark procedure for thematic analysis.

**Result:**

The analysis revealed three themes in family members’ experiences of being a caregiver to a cancer patient throughout a cancer trajectory. These were: (1) From the time of diagnosis—overwhelming and uncertain; (2) During and after treatment—invisible and not involved; (3) Throughout the cancer trajectory—an emotional roller coaster.

**Conclusion:**

The results indicated that the family members felt invisible and not involved and they experienced being a caregiver throughout the cancer trajectory as an emotional roller coaster. Our empirical findings thus indicate that in cancer care, family perspectives are yet to be implemented in daily practice. This is in contrast to explicit goals in current health policies underlining support and involvement of family members as a core aspect in cancer care.

## Introduction

Health care systems consist of both formal and informal caregivers. Formal caregivers are defined as paid health personnel who have had training and education in providing care. Informal caregivers are defined as relatives and family caregivers of the patient, usually without payment (Johns Hopkins Medicine, 0000). In the research literature, many terms for informal caregivers are used, such as family members, family caregivers, next of kin, and relatives (used as synonyms in this article). In the literature, there is no single or universal definition of a “family caregiver” (Williams, 2018) or “caregiver” (Romito et al., 2013). In line with the understanding expressed in health and patient laws, we chose to define the patients’ next of kin or relatives as the person(s) whom the patient him- or herself names to take this role. The person(s) defined by the patient as next of kin has different rights than those of other relatives with regard to consent, information, access to medical records and complaints (Lov om pasient- og brukerrettigheter, 2021).

Recognizing the role and competence of relatives as important has been documented in several national (Skirbekk et al., 2018; Søvde et al., 2019; Tarberg et al., 2019) and international (Moreno-González et al., 2019; Romito et al., 2013; Sklenarova et al., 2015; Sun et al., 2019) studies and in public policy documents. Family caregivers play a vital role in the direct care and support of patients with cancer, and the welfare state is completely dependent on them (Dieperink et al., 2018).

Family-centred care (FCC) is an important model as it improves the quality of care (Berger et al., 2014), and benefits both patients, family members and healthcare professionals (Park et al., 2018). The FCC model identifies four core concepts: dignity and respect, information sharing, participation, and collaboration (Johnson & Abraham, 2012). These core concepts recognize that health care improves when patients and their families have their perspectives and beliefs incorporated into care, when they receive accurate and level-appropriate information, and when they are encouraged to participate in decision-making for their own care and to collaborate beyond their own care to improve policies, programmes, facilities, research, and education (Johnson & Abraham, 2012). However, little standardization or system-level implementation of family-centred care models to integrate and support families has emerged in adult oncology care settings (Reblin et al., 2021).

Policy documents often emphasize certain aspects over others when it comes to cooperation with relatives. Responsibility, duty and performance of work are emphasized, while authority and rights are omitted (Jenhaug, 2018). Most health care systems do not have a formal procedure or standardized mechanism for integrating and supporting caregivers (Reblin et al., 2021). Healthcare professionals have a duty of confidentiality concerning the patient’s sickness or other personal circumstances, but they should create the conditions for relatives to contribute and be involved as far as the patient wants this (Lov om pasient- og brukerrettigheter, 2021). Most patients want to involve their relatives (Dieperink et al., 2018), but in some cases relatives do not represent a resource for the patient (Seibæk et al., 2012). However, healthcare professionals are obliged to give relatives general information and guidance about contact details for healthcare services, procedures, legislation and rights, services available to relatives, organizations for users and relatives, etc (Norwegian Directorate of Health, 2018).

Cancer can have major consequences for caregivers as well as patients, and health care systems often overlook the needs and role of caregivers (Kent et al., 2016). Caregivers are often ill-prepared to perform the many tasks needed to care for the patient (Sun et al., 2019). Consequently, for family members, the role of a caregiver often comes with substantial distress and burden (Kent et al., 2016), which can lead to lasting negative health effects for both the caregiver and the patient (Kim et al., 2015). Nevertheless, it is possible to have positive experiences, or post-traumatic growth, during the cancer trajectory. The term post-traumatic growth (PTG) refers to the cognitive process through which those who have experienced a traumatic event positively find meaning in the event, such as (Tedeschi & Calhoun, 1996).

In every phase of the cancer care trajectory, family caregivers provide patient care, such as symptom management, meals and nutritional assistance, supervision of treatments, emotional and physical support, coordinating care, monitoring using electronic devices and communication with providers (Kent et al., 2016; Schulman-Green et al., 2021).

In this study, it was the patients who defined who their relatives or family members were, and the family members could be a blood or adoptive relative, friend, neighbour, or adult child who maintained a separate home. At least one of the family members in each of the families interviewed was next of kin in terms of the legal definition in (…). Furthermore, the term “cancer trajectory” will be used for the phase from diagnosis until around one year after all chemotherapy and radiation treatment.

To date, many studies have focused on caregivers’ unmet needs (Girgis et al., 2013; Sklenarova et al., 2015; Wang et al., 2018) and burden (Essue et al., 2020; Tan et al., 2018). In this study we wanted to focus on family members’ experience of being a caregiver over time. Knowledge about health and illness experiences can be used to improve the quality of healthcare services (Ziebland et al., 2013) and to develop procedures and routines for how to take care of caregivers in health care. To the best of our knowledge, the cancer care literature includes very few longitudinal studies about family members’ experiences of being a caregiver throughout a cancer trajectory, from diagnosis until around one year after treatment. Therefore, to expand the existing knowledge base about caregiving in cancer care, we have studied the family members’ experiences at three time points during the cancer trajectory. The aim of this study was to explore family members’ experiences of being a caregiver throughout such a cancer trajectory.

## Method

### Design

The study used a longitudinal qualitative design in order to gain deeper knowledge and describe family members’ experiences of being a caregiver throughout the cancer trajectory. This design is suitable when data are collected at more than one point in time with the same individuals (Polit & Beck, 2008), to explore experiences over time (Malterud, 2017).

### Context of the study

Norway has a semi-decentralized health system with four regional health authorities (RHAs). The RHAs are responsible for specialist care and the municipalities are responsible for primary care and social services (Saunes et al., 2020). This study was conducted in one Norwegian health region consisting of 46 municipalities with their separate primary health services, and one common hospital for specialist health care. The hospital is organized into seven local hospital units with a catchment area of approximately 300 kilometres.

### Recruitment and sample

The recruitment was done through an outpatient clinic; the doctor briefly informed the patient about the study and asked them if they wished to speak with the first author, immediately after the consultation. Those who agreed got oral and written information about the study that they could discuss with their family. The informants possessed certain characteristics and qualities in line with the study’s objective (Malterud, 2017). It was the patients who recruited the family members. The inclusion criteria for the patients who participated in the main study were that they had a confirmed cancer diagnosis, spoke and understood Norwegian, were competent to give informed consent and over the age of 18.

The inclusion criteria for family members were that they spoke and understood Norwegian, were competent to give informed consent and over the age of 16. There were 13 family members included in the study. Table 1 gives an overview of the characteristic of the participant and other elements that are central for the study.

**Table 1.** Characteristic of the participants and other central elements.

	Patient diagnosis	Relationship to the patient	Next of kin	FMs’ age	Living withthe patient	Interview		
						1	2	3
Family 1	Lung	Son	Yes	41–50	No	x	x	x
		Son	Yes	41–50	No	x	x	x
Family 2	Breast	Partner	No	51–60	No	x	x	x
		Daughter	Yes	21–30	No	x	x	x
Family 3	Breast	Husband	Yes	41–50	Yes	x	x	x
		Friend	No	41–50	No		x	
Family 4	Breast	Aunt	Yes	51–60	No	x	x	x
		Friend	No	51–60	No	x	x	x
Family 5	Breast	Son	Yes	41–50	No	x		x
		Daughter	Yes	41–50	No	x	x	
Family 6	Breast	Husband	Yes	51–60	Yes	x	x	x
Family 7	Breast	Partner	Yes	51–60	Yes	x	x	x
		Daughter	Yes	21–30	No	x	x	

### Data collection

Individual in-depth interviews were used as a method for data collection. Each family member was interviewed once, twice or three times (see Table 1). The reason why not everyone was interviewed three times is either that they were unavailable during the relevant period or that they did not respond to the inquiry. Due to the small sample, we chose to include everyone, even if they had not participated in more than one interview. The interview was based on a thematic interview guide consisting of one main question and four topics related to the research question. The main question in the first interview (before treatment) was: “Can you tell me your story, from the time you suspected something was wrong until now?” The main question in the second (during treatment) and third (about a year after treatment) interview was “Can you tell me your story, from what happened since our last talk?” The four topics were the same in all interviews: everyday life, information from the hospital, family and surroundings, quality of life, and follow-up during the waiting phase. The interview guide was tested on two user representatives and then developed with input from them. The material was digitally recorded by the first author and transcribed verbatim by the first author and a professional transcriber. The quotes in this article have been translated from Norwegian to English by a professional translator.

The interviews were conducted by the first author from June 2018 until December 2020. There were no relationship between the interviewer and the participants. Data were developed from open individual interviews. The interviewer allowed the family members to talk freely. In most of the interviews, the family members covered the topics themselves in their history, without the interviewer needing to ask the questions. The family members chose when and where the interviews should be conducted. The patient was never present during the interviews. Twenty-two took place in their home, two took place in an office at the hospital and ten via Skype. Each interview lasted from 12 to 60 minutes (average time 33 minutes).

### Ethical considerations

This study was approved by the Centre for Research Data (NSD) (project number 51,466), and was conducted in accordance with the Declaration of Helsinki (Saunes et al., 2020). The informants received oral and written information about the study before giving their written consent. They were told that they could withdraw from the study at any time. Data in the study were stored in accordance with regulations.

### Data analysis

Thematic analysis was chosen as a method to analyse the transcripts of the in-depth interviews. The analysis was inspired by the Braun and Clarke (Braun & Clarke, 2006) six-phase process. First, the first author read and reread the transcript material to become familiar with the content. Then, the same author imported the transcript material into computer software (NVivo V.12) to identify an initial set of codes. Next, the codes were organized into potential themes. At this stage, all authors reviewed the codes in relation to the potential themes to ensure that they reflected both the associated coded extracts and the entire data set. To ensure this, the authors worked back and forth between the codes, the entire data set, and the potential themes. In a series of meetings between the authors, the potential themes were discussed and renamed. The potential themes were then summarized into three main themes that reflected the most important experiences as perceived by the family members throughout the cancer trajectory. An example of the analysis process can be found in Table 2. Direct quotes from the family members (translated into English by a professional translator) are used to illustrate the findings.

**Table 2.** Example of the analysis process.

Data extract	Code	Theme
“it was not confirmed that it was not lymphoma, because I felt terrified of that when he started talking about lymph, because I’ve heard dreadful stories about that”	Was terrified when the doctor talked about lymph	The time of diagnosis—overwhelming and uncertain
“then it’s good to talk about it, because the fear comes instantly, then it gradually gets better and better as you get more information and you realize that those around you know what they are talking about and are experts”	The fear comes first, then it gets better	
“but it’s not so dangerous, but you’re sort of scared of that big word cancer then, hard to say it out loud”	The word “cancer” is hard to say out loud	

### User involvement

Two user representatives were involved when we discussed both our aims and interview guides, and our early findings. Both had experiences of having cancer and being a caregiver for someone with cancer.

## Findings

**Figure 1.**
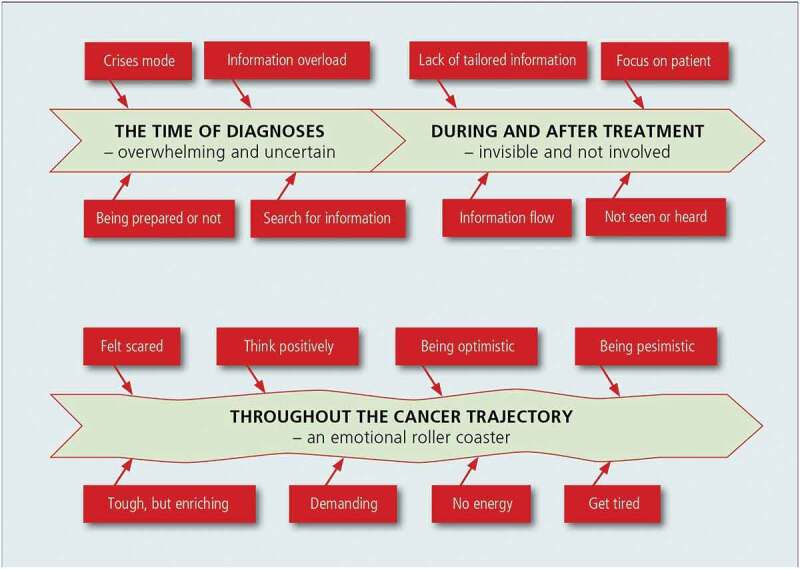
Overview of the findings presented as themes with experiences.

The aim of this study was to explore family members’ experiences of being a caregiver throughout a cancer trajectory, from diagnosis until around one year after treatment. Findings in this study illuminate how family members experienced that being in this journey with the patient began with the shock and uncertainty of the diagnosis and continued through the stress of managing side effects of treatment to survivorship and how this would affect their own life. The different phases of the cancer trajectory were experienced differently by the family members, such as if the patient had side effects from treatment or not. Three themes emerged from the analysis concerning family members’ experiences of being a caregiver throughout a cancer trajectory in Norway from diagnosis until around one year after treatment: from the time of diagnosis—overwhelming and uncertain; during and after treatment—invisible and not involved; and throughout the cancer trajectory—an emotional roller coaster (see Figure 1).

### The time of diagnosis—overwhelming and uncertain

The family members experienced the period before the start of treatment as overwhelming and uncertain. The fact that their loved ones did not appear to be ill made it overwhelming and difficult to deal with the actual cancer diagnosis.

If it was a disease that you could actually see, it would have had an effect on you in your everyday life, but in practice it doesn’t yet do that, so in that way it is very strange. Because there’s (…) no difference except that we know something is going to happen (F1-1)

Family members who had participated in the consultation where the diagnosis had been communicated experienced that the health personnel only talked to the patient, and that they had to speak up to be heard. One female family member experienced it like this:

They did not address me, it was her they talked to, then I had a chance to ask a couple of questions – for them, I was not there as a family member. That wasn’t the impression I got, at least. (F6-1)

Furthermore, they commented that the information was informative about the treatment itself, but that there was no involvement. A female family member put it this way: *They do not outline any choice, there is no involvement somehow (…) it is difficult, think it would have been easier if one had become more involved (F6-1).*

Several of the family members expressed their uncertainty about how the cancer diagnosis would affect the ill patient and their everyday life. They had many questions after being told about the diagnosis but did not know where to turn with their questions. Several of them chose to search the internet, without knowing which sites were reliable and accurate for obtaining information about the diagnosis. Fear was also highlighted as a response to not knowing the severity of the cancer diagnosis. A female family member told how she handled this uncertainty:

You have to choose to shut it out, if, in a way, how should I put this, you’re going to manage to survive, perhaps what I did was a bit like that. You block it out, right until you get the answer in black and white (F4-1)

Some of the family members had previous experiences with cancer in a close relationship and described this as having advantages and disadvantages. The advantage was that they knew what it meant to live with a serious illness, and that they had some overview of how the process worked and what they could expect. A female family member described the experiences as follow: *you are never prepared, but I feel that I am more prepared in relation to knowing what it is like to be ill then (F6)*. One of the disadvantages of having these experiences was expressed by a female family member as follows: *[name of the patient] had to be rebuilt time and time again. It’s the roller coaster you dread (…) And you are emotionally part of that roller coaster (F4)*. Some also expressed this experience as a strength and as providing a sense of security.

### During and after treatment—invisible and not involved

Family members had no experiences of offers from health care personnel to provide information and education about the symptoms of cancer, its treatment (chemotherapy and radiation therapy) and consequences, through the cancer trajectory. Furthermore, they received no information about support groups or other activities that were tailored for them as family members. The lack of information and guidance from the health care personnel created feelings of frustration and concern about their own health.

And it’s such a, I think … for many family members it’s probably like this, that you keep going, you are there for the others, you’re supposed to be the big strong one. That’s OK for a while, but not for the long term. And … no, there should have been a system that responded to that … I think. (F13-2)

The family members commented that the health care was principally there for the patients, and not for them. They said that this was understandable, and that they did not have any expectation of being involved as caregivers. Nonetheless they expressed a need to be seen and acknowledged. One of the family members was a health professional and said that it was a useful experience to “be on the other side” and see the importance of taking relatives seriously.

You really get a better picture of how important it is to be met as a family member. I do understand that in a nurse’s everyday life, it is easy to just focus on the patient in a way. But after all you do not know who the family members around are, or what kind of thoughts (…) they have, so what (…) they think (…) is very important then. And at least when you have sort of been there yourself, then (…) you get a better picture of it (F12-2)

The family members expressed that their patient had to deal with different health practitioners and different treatment places, and that they were unsure of how much information had been lost in the transition. They experienced that the GP was not up to date. One male said: *I’m on the point of saying that it is a system error* (…) *Then the potential for improvement there is enormous* (F3-2). This experience led them to be uncertain about the flow of information, and if the patient was receiving the best available treatment.

It doesn’t seem like there’s a very good flow of communication between these different hospitals. You’d think that now that we have so much data and so on, it should be possible to have everything in one place. And then it shouldn’t be so difficult to just go in and see: What have you been informed about? (…) And that nothing is sent to the GP, I can’t understand that either. (F8-2)

Some of the family members also commented that the information flow seemed random and dependent on the individual, and they felt like an extension of the patient, and not like an individual with their own needs and feelings. Furthermore, they expressed thoughts concerning the routines in involvement of family members and asked themselves questions like “do we have any rights?”

No. Well, I have been thinking a bit about what, in a way, the hospital’s point of departure is. What kind of information do they give? I mean, do they have any routines for (…) contacting family members? (…) And if it is different if the person is sick? But I guess I’ve come to the conclusion that there’s no such thing (F2-3).

Furthermore, they asked themselves who was responsible for keeping everyone up to date. As one female family member said: *It is … and the closest family members are not supposed to be health professionals either. That’s not their job (F13-2).*

Several family members expressed that being in a standardized cancer pathway had both benefits and weaknesses. The benefits were that the medical aspects, such as general information about examination and treatment, were well taken care of, and that the time from diagnosis to the start of active treatment was short. The weaknesses were that it did not seem to be a system for individual care and it provided no information about how a cancer diagnosis could lead to side effects other than physical effects.

In my experience it’s very good as far as the complete medical overview is concerned, relating to the procedure itself and illness (F10-2) and then it is very limited when it comes to the psychological side (F10-3)

### Throughout the cancer trajectory—an emotional roller coaster

Several family members commented that the word cancer triggered many different emotions and reactions. Some said that they felt scared and it was difficult to say the word *cancer* out loud, and fear was the first feeling they experienced.

Who doesn’t get terrified by that? You think that you are going to die straight away, and that’s the way it is, (…) then it’s good to talk about it, because the fear comes straight away, then it gradually gets better and better as you get more information and you realize that those around you know what they are talking about and are experts (F6-1)

Others emphasized that they chose to think positively about what would happen in the future. A male family member had read online about the prognosis for the diagnosis, where he commented: *it said something like that after five years, 20–25% are still alive. It is clear that something positive can be read (F1-1)*. Furthermore, he said that he was born optimistic and not pessimistic. Several commented that being optimistic and not pessimistic was important to them to keep their mood up, but at the same time, they had to be realistic. A male family member expressed his optimism as follows: *(…) you do not paint the devil on the wall (…) (F3-1)*. Some of the patients developed side effects from the treatment. The family members described this experience as demanding, but enriching at the same time. It was hard to see the patient’s transformation from an independent individual to somebody who was completely dependent and helpless.

Yes, it’s that tough part with chemo, then, for her. That she has been a bit washed out after these first four intense treatments. Then we’ve had a whole week when she has been absolutely wiped out, really. So it’s been hard to see her feeling so ill. That’s the worst, after all (F5-2)

It was of great importance for the family members to give an impression of strength and security around the patient. They expressed that this affected them emotionally and they did not know where to turn with their thoughts. Furthermore, they said that they put their own needs aside to take care of the patient, and they expressed a concern about lack of time and energy to engage in self-care and care of others.

She just lay on the couch and had no energy to do anything, that was very tough (…) you feel like you want to be there all the time, but at the same time (…) I sensed that I needed to try to take some distance (…) But then you get very tired yourself. But it’s mainly in my head (F4-2)

In one of the families the patient and her husband ran a business together. Combining the role of employer and spouse was perceived as demanding. Employers are summoned to dialogue meetings with the patient and social security services to discuss the work situation of the patient. In these meetings you are met only as an employer and not as a spouse.

Obviously, it affects you, because you just have to solve things, and you get more things to solve (…) So you use quite a lot of your remaining resources as well. So then it’s clear that you are not as good, neither as a leader nor as a spouse nor as a dad (F10-3)

Family members described going through a hard period as not only tough, but also enriching. They became more aware of what they spent their time on, and saw things in a slightly different perspective. Furthermore, they perceived being able to provide help and support as positive.

This thing about switching roles. She’s always been the one who’s been everywhere, who’s been the strong, positive, super cheerful woman. And to see her so vulnerable and so weak now – that has been pretty tough. But at the same time, it has been good for me to be able to give something back. So, now I really feel like I could have supported her in return, then (F13-2)

The family members reported that being in this emotionally phase brought them closer to each other, and that this provided an opening to talk about existential topics. This was experienced as good, despite the tough phase they were going through.

That we have actually sat and talked so openly about death has brought us even closer to each other (…) incredibly lucky to have such a friend that you can share everything with, and who you know is there whatever happens (F13-2)

Family members expressed that both positive and negative experience, from the time of diagnosis to after treatment, has left them with new perspectives on life such as

More mindful about keeping in touch (F1-3) – Don’t worry unnecessarily (F2-3) - Once something happens, you see how fragile everything is. And then you put more emphasis on the small things in everyday life (F7-3) - Appreciate the family more (F9-3) - If we are healthy here in this world, we never have any problems. It’s as simple as that (F11-3)

## Discussion

Our results are interesting from a family centred care (FCC) perspective and can inform ongoing debates on next of kin involvement in healthcare. In January 2017, the “Relatives’ Guide” was published by the Norwegian government. This is similar to initiatives in other countries, such as Denmark (Danish Health Authority, n.d.) and Canada (Canadian Caregivers Coalition, 2013). One of the main goals for the “Relatives’ Guide” was that relatives should become involved in the health and care service for the benefit of the patient and relatives, if the patient wishes this (The Norwegian Health Directorate, 2018). Despite the good intentions of government documents, such as guidelines and strategies, which describe how to involve and support relatives, findings in this study show a discrepancy between policy intentions and family members’ experiences. Health personnel may have the best intentions, and there may be several reasons why they do not meet the interests of family members. Some of the reasons may be insufficient resources and knowledge about family member’s needs, routines and strategies (Ekstedt et al., 2014). Supportive relationships between the health services and caregivers seem to be important to facilitate more effective communication (Reblin et al., 2021). However, traditional training in communication skills has focused on aspects of patient and provider communication, and not on supportive communication with caregivers (Laidsaar-Powell et al., 2016).

The study results revealed that health care services on all levels lack routines for mapping family member’s needs for necessary and desired information and involvement, in order to be able to support the patient and themselves. Furthermore, the findings show that family members did not get any information at all that was tailored to relatives’ needs, and this also applied to information about their rights and benefits and existing services. This finding was also reported by Birtha and Holm (Birtha & Holm, 2017), where the family members complained that it was complicated and time-consuming to find all the information themselves. Family members are flooded with information about diagnosis and about how it will affect them as caregivers. Therefore, it will be important for health personnel to assess the caregiver’s needs, inform about reliable and trustworthy sources for obtaining information, and facilitate family support as far as possible (Mastel-Smith & Stanley-Hermanns, 2012).

Our findings show that early in the cancer trajectory family members did not quite know what they needed, as the diagnosis itself was overwhelming to digest. This finding was also reported by Nissim, Hales (Nissim et al., 2017), where the caregivers reported that they were still in a state of shock and not ready to absorb and embrace extensive information at the time of diagnosis. It is more likely that caregivers will be more effective and less likely to feel overwhelmed if both they and their patient are included as members of a care team, working towards a common goal (Berry et al., 2017). Uncertainty was also highlighted as a reaction early in the cancer trajectory. The uncertainty was related to the diagnosis and everyday life. A variety of studies have found that lack of knowledge increases uncertainty (Arias-Rojas et al., 2020; Guan et al., 2021), and leads to loss of control (Wang et al., 2018). To manage uncertainty, informational support is one of the key components (Guan et al., 2021).

Findings in this study show that family members do not have a great need for emotional and psychological support for themselves, at the time of diagnosis, but that this need changes during the cancer trajectory. This is in line with a previous study (Girgis et al., 2013) showing that after 12 months (from the diagnosis), caregivers seemed to need less cancer-related information and more support with looking after their own health. A study conducted by Sato, Fujisawa (Sato et al., 2021) emphasized that tailored care and support for the family caregiver is needed immediately after diagnosis. In contrast, we found that the period immediately after diagnosis was characterized by patients and next of kin feeling overwhelmed. We can only speculate about the reasons for these diverging results: nevertheless we find it plausible that our in-depth investigations through multiple interviews over time allowed the informants to share more nuanced experiences and feelings than the written questionnaire used in the Fujisawa study (Sato et al., 2021).

As might be expected, our study also indicates that family members’ need for information and emotional support is greater where the patient has a higher symptom burden. Caregivers who are in good emotional and physical health are more likely to provide emotional, practical and self-management support for the patient (Litzelman, 2019). Despite this, our study shows that current cancer care services seem to imply expectations that families should support patients, but do not include functions/systems/routines for providing any practical or psychological support to meet these expectations. This is in line with other studies where caregivers reported a lack of awareness of formal services (Reblin et al., 2021) and being generally invisible to the health system and rarely identified as care partners (Hassankhani et al., 2019). Caregivers continue to report feeling unseen, being dismissed as insignificant in clinical encounters, and having unmet needs that range from dealing with their own emotional distress to information about the diagnosis (Litzelman, 2019). The unmet needs of informal caregivers are often ignored and excluded from healthcare planning (Halkett et al., 2015; Sealey et al., 2015).

Recent research has tended to focus on the negative experiences and outcomes, such as unmet needs and burden, of the caring role. Cancer is a traumatic experience for both the patients and the family members. Nonetheless, positive consequences may emerge through the pain from traumatic experiences into positive and meaningful growth (Snyder et al., 2020). Studies by Matthews (Matthews, 2020) and Nouzari, Najafi (Nouzari et al., 2019) found that PTG for cancer survivors and family members took the form of improvement in their relationship, and that the level of hope had an effect on PTG. This is similar to our findings, but our family members also emphasized that the strengthened relationship created opportunities for talking about existential topics like death and life.

## Limitations and strengths

A possible limitation of the study may be the small sample of family members that participated and that they were only from one part of (…). Nevertheless, the findings in this study should contribute to a richer understanding of family members’ experiences of being a caregiver when a family member is diagnosed with cancer. To increase the credibility of the study, all family members were interviewed by the same interviewer (M.S.). The interviewer was a registered psychiatric nurse with extensive experience and knowledge in talking to people in vulnerable situations. To further strengthen the trustworthiness of the study, two user representatives contributed throughout the process of designing project descriptions and interview guides.

## Conclusions

Overall, this study expands knowledge about experiences that are highlighted throughout the cancer trajectory, as perceived by family members. The results indicated that there is a discrepancy/gap between what policymakers describe regarding how to support and involve relatives in cancer care and how the family members experience this role. The family members felt invisible and not involved, and they experienced that being a caregiver throughout the cancer trajectory was an emotional roller coaster.

Yet our current practice and healthcare systems do not seem to have any standardized routines and strategies to identify caregiver’s needs or to implement policymakers’ intentions in clinical settings. Broadening the current focus from patient-centred to family-centred healthcare could be one solution to achieve this goal.

Further studies are important in order to develop and implement a family-oriented model that encompasses family-centred care to enhance the family as a unit. Family members are a valuable resource and should be involved in an early stage in cancer care.
